# Evaluation of Simulated Shelf-Life Conditions for Food Service Applications on Chicken Tenderloins

**DOI:** 10.3390/ani11072028

**Published:** 2021-07-07

**Authors:** Laura E. Yoder, John G. Rehm, Hunter R. Smith, Daniel A. Tigue, Barney Wilborn, Amit Morey, Christy L. Bratcher, Eugene Blythe, Jason T. Sawyer

**Affiliations:** 1 Department of Animal Science, Auburn University, Auburn, AL 36849, USA; ley0010@auburn.edu (L.E.Y.); jgr0012@auburn.edu (J.G.R.); hzs0101@auburn.edu (H.R.S.); dat0002@auburn.edu (D.A.T.); wilbobs@auburn.edu (B.W.); 2 Department of Poultry Science, Auburn University, Auburn, AL 36849, USA; azm0011@auburn.edu; 3 Department of Animal Science, Texas Tech University, Lubbock, TX 79430, USA; christy.bratcher@ttu.edu; 4 College of Agriculture, Auburn University, Auburn, AL 36849, USA; blythek@auburn.edu

**Keywords:** chicken, marination, microbiology, shelf-life

## Abstract

**Simple Summary:**

Poultry products are popular meat products in the United States for both retail and food service sectors. Food service operators typically obtain food products in bulk as they utilize products quickly and at a high volume. Typically, chicken arrives to food service operators frozen in bulk packaging and is thawed or slacked by storing it in refrigerated temperatures (2 °C to 4 °C) to be used over several days while maintaining its acceptability for cooking and serving to consumers. Ensuring a product is safe to consume is the most important factor in the food industry. This study measured the microbial growth on marinated chicken tenderloins that were aged after slaughter, bulk-packaged, frozen, then slacked for 132 h. At no time during the slacking period did any samples reach the limit (6 log) of unsafe microbial growth. Psychotropic bacteria grew at each sampling time and the tenderloins aged for 4 and 5 days post-slaughter surpassed all other treatments. As no samples surpassed the spoilage threshold, it is suggested that slacking is a safe method of thawing chicken tenderloins for up to 8 days post-slaughter.

**Abstract:**

The objective of this study was to validate the shelf-life of marinated and frozen chicken tenderloins. Treatments were randomly assigned to the age of the tenderloins post-harvest, days aged (DA): DA4, DA5, DA6, DA7, and DA8. Microbial analyses were used to analyze the growth of aerobic, psychotropic, and lactobacilli bacteria to assess the shelf-life of bulk-packaged chicken tenderloins. Tenderloins were sampled fresh, then vacuum tumbled in a marinade. After marination, the tenderloins were sampled with the remaining tenderloins packaged and frozen (−25 °C). After freezing the chicken tenderloins were slacked in a refrigerated cooler (2.2 °C) for up to 132 h (h) and sampled at 36 h, then every 24 h following. After marination, each treatment significantly (*p* < 0.05) decreased in aerobic and psychotropic counts except DA4. During slacking, no treatment crossed the threshold of 10^6^ CFU/mL (Log 6) set for this study. Though none crossed the threshold, treatments DA4, DA5, and DA6 had significant (*p* < 0.05) increases in aerobic bacteria after 7 days of age. The psychotropic bacteria continuously grew at each sampling period, with DA4 and DA5 surpassing the other treatments (*p* < 0.05) at 108 h and 132 h reaching 105 CFU/mL. Every treatment remained below the spoilage threshold, suggesting that this method of storage is suitable for chicken tenderloin shelf-life.

## 1. Introduction

The consumption of chicken products in the United States has dramatically increased to approximately 240 kg per person since 1959. With such stark increases in consumption, it has been reported that over 42% of chicken consumed is sold through a food service outlet [1], where many steps occur to create this product for consumers. Moreover, it has been reported [2,3] and is widely known that excessive handling of meat products by consumers and food service providers, specifically poultry, through the processing and manufacturing stages results in a greater likelihood that microbial loading can occur [4,5]. The testing of raw and finished poultry products for initial microbial load of a portioned product from start to finish can aid in developing prediction models for meat manufacturing companies. Research has concluded that microbial growth (10^6^ to 10^7^ CFU/g) can create signs of spoilage organism development, including but not limited to off odors and slime production [6,7]. Aerobic, anaerobic, and psychotropic microflora are common reasons for a meat product to spoil [2]. A common spoilage microorganism in the meat industry, *Pseudomonas*, is a gram-negative psychotropic bacteria which has been reported to grow in cold storage environments, including in coolers or refrigerators [8]. Additionally, gram-negative bacteria require higher water activity for growth survival, which can be found in poultry products [4]. Lactic acid bacteria (LAB) is another common category of bacteria which can be identified on food products including meat and poultry. By using microbial sampling methods, the deterioration of poultry meat products can be conducted to assess raw materials and the packaging methods used in distribution channels. Flat packaging has been utilized in the food service industry due to its speed, ease, and ability to control package volume. In the use of flat packaging within the poultry industry, the product is typically frozen then thawed in refrigerated temperatures (2 to 4 °C) for several days, often referred to as slacking. Additionally, the majority of the poultry processed in the United States is marinated in a solution containing water, salt, and phosphate to increase water holding capacity to promote a juicier product [6]. This is achieved by injecting the solution and possibly tumbling a product under vacuum, which aids in the distribution of the solution through the product and allows for protein extraction [6]. Thus, the objective of this study was to validate the shelf-life of vacuum-tumbled marinated chicken tenderloins after frozen storage and slacking through the analysis of microbial growth.

## 2. Materials and Methods

### 2.1. Raw Materials

Fresh chicken tenderloins were obtained 48-h post-mortem, hand trimmed, weighed, and sorted from a commercial processing facility (Foundation Food Group; Gainesville, GA, USA). After portioning, a total of 680.4 kg of chicken tenderloins (226.8 kg per replication × 3 replications) were placed inside a plastic liner and then divided into 150 qt insulated coolers (Igloo, 105.75 cm × 47.48 cm × 51.44 cm, Katy, TX, USA). Ice was placed in the cooler prior to the addition of the tenderloins, with a plastic liner used to ensure no moisture migrated from the ice to tenderloins. A ThermaData series II Temp Logger T2C (2 Ext. Removable Probes, American Fork, UT, USA) temperature data logger was inserted into each cooler in two different adjacent locations to monitor product temperature during transportation. Chicken tenderloins were transported to the Lambert-Powell Meat Laboratory at Auburn University. Upon arrival, tenderloins were allocated randomly to 3 replications (226.8 kg per replication) and kept in dark storage for 48 h at 2 °C.

### 2.2. Treatment Allocation, Marination and Packaging

Chicken tenderloins (226.8 kg) were weighed and randomly assigned to 1 of 5 treatments (45.36 kg) based on days aged (DA) post-harvest: DA4, DA5, DA6, DA7, or DA8. Each treatment had 2.27 kg removed for microbial analysis after an initial assignment. On each of the corresponding days post-harvest (4, 5, 6, 7, or 8), each treatment group (43.09 kg) was subjected to marination by vacuum tumbling in a proprietary blend (1.64 kg) including: water, salt, modified corn starch, and monosodium glutamate (MSG) for 6 min at 4 rpm. After marination, 2.27 kg of tenderloins were again removed for microbial analysis. The remainder of the chicken tenderloins in each treatment group (40.82 kg) were packaged into blue plastic bags (C and E Supply LLC, 13 × 20 + 1.5″ LIP Blue Bag) (2.27 kg/bag) and pressed flat by hand. Flat packing was achieved by pushing any remaining air out of the bag and folding the top flap of the bag over with no actual seal. This entire process was performed with 3 replications.

### 2.3. Product Storage and Slacking

Each blue bag was placed into a blast freezer (−25 °C) where they remained for 8 d until their treatment slacking processes began. After 8 d of storage in the blast freezer, each treatment was removed and placed into a walk-in cooler (4 °C ± 2 °C) for 36 h to begin the slacking process. After 36 h, three (*n* = 3) blue bags were removed and used for analysis, then every 24 h following (up to 132 h) 3 blue bags were removed for analysis, to simulate the food service operator thawing specifications. When blue bags were removed from the cooler they were subjected to microbial analysis.

### 2.4. Microbial Analysis

Chicken tenderloins (*n* = 2) were aseptically removed from each blue bag immediately after removal from the refrigerated cooler using a modified procedure of the American Public Health Association [9]. Tenderloins were placed into a Nasco Whirl-Pak filter bag (55 Oz. Filter Bag 7.5″ × 12″, Nasco, Fort Atkinson, WI, USA) with 50 mL of phosphate buffered salt (PBS) and hand massaged for 1 min. Following massaging, 1 mL of the PBS solution was extracted from the filter bag with a serological pipette tip and placed into a dilution tube containing 9 mL of PBS to create serial dilutions. Each dilution was mixed using an analog vortex mixer (VWR International, Radnor, PA, USA). After serial dilution, 100 μL was extracted from each dilution tube and the filter bag and placed onto 3 media types: aerobic plate (Difco™ Plate Count Agar; Becton, Dickinson, and Company; Tempe, AZ, USA); psychotropic plate (Difco™ Plate Count Agar, Becton, Dickinson, and Company; Tempe, AZ, USA); and de Man Rogosa and Sharpe agar (Difco™ Lactobacilli MRS Agar; Becton, Dickinson, and Company, Tempe, AZ, USA). Aerobic and psychotropic plates were incubated in a Jeio Tech, Inc. incubator (Model IB-05G, Jeio Tech Inc., Woburn, MA, USA), with aerobic plates incubated at 37 °C for one day and psychotropic plates incubated at 8 °C for 7 days. MRS plates were incubated at 37 °C for two days in anaerobic chambers (MGC AnaeroPack^®^ System, Rectangular Jar 7.0 L; Mitsubishi Gas Chemical Co., Inc., Chiyoda, Tokyo, Japan) which contained two oxygen scavenger packs (GasPak™ EZ; Becton, Dickinson and Company, Sparks, MD, USA) to reduce free oxygen. Each sample had 3 serial dilutions plated per plate type for a total of 9 plates per sample. After the incubation period, plate colonies were counted on a Reichert Quebec Darkfield Colony Counter (Depew, NY, USA) and recorded. The best plate was taken from each sample and then converted to CFU per mL of rinsate.

### 2.5. pH Analysis

On each day of sampling, pH was measured using a probe style-pH meter (H170 Hach pH meter, Hach, Loveland, CO, USA), calibrated each day using a standard 4.0 and 7.0 buffer solution prior to collecting microbial and instrumental color samples. Chicken tenders (*n* = 2) were removed from their respective package and a stainless-steel probe was inserted into the geometric center of each tender. The average reading of three readings from each tender within each treatment across all sampling days was recorded.

### 2.6. Instrumental Surface Color Analysis

Fresh surface color was measured using the Commission International de’Eclairage (CIE) spectrum for lightness (L*) using a HunterMiniscan XE Plus (MSXP-4500C; Hunter Laboratories, Reston, VA, USA). Color measurements were measured in duplicate on the surface of the chicken tenders and the mean value was recorded for each tenderloin.

### 2.7. Statistical Analysis

For this study, 3 replications were conducted with each microbial plate type being plated 3 times per replication. Microbial data was converted to log_10_ CFU/mL rinsate prior to statistical analysis. Media type was considered a repeated measure and days aged and slack time were fixed effects. The data were analyzed using a PROC GLIMMIX of SAS 9.4 (SAS Institute, Inc., Cary, NC, USA) and LS Means were separated using the Tukey–Kramer adjustment with α = 0.05.

## 3. Results

### 3.1. Aerobic Microbial Analysis Results

Results from the microbial analysis utilizing aerobic plates are presented in Table 1. Fresh tenderloins from DA4 were the only treatment to significantly (*p* < 0.05) decrease in aerobic plate count (APC) values by 0 h, however those values increased by 36 h and were similar to fresh tenderloin values. Chicken tenderloins from DA4, DA5, and DA6 when sampled fresh had significantly (*p* < 0.05) lower APC values than DA7 and DA8. Although DA7 and DA8 both started with values significantly greater than all other treatments, they were the only treatments with significantly (*p* < 0.05) lower APC values at 132 h than their fresh APC values. After marination, all treatments had numerically lower APC values, however only DA4 had significantly (*p* < 0.05) lower values at 0 h. By 36 h slack time, DA4 and DA5 had significantly (*p* < 0.05) lower APC values than all other treatments, however both treatments significantly (*p* < 0.05) increased from 36 h to 60 h. By 132 h slack time, DA4 had the highest APC values significantly (*p* < 0.05) greater than all treatments other than DA7, however all other treatment were not statistically (*p* > 0.05) different from each other at that time.

### 3.2. Lactic Acid Bacteria Microbial Analysis

Results from lactic acid bacteria (LAB) microbial analysis are presented in Table 2. Tenderloins from DA4, DA5, and DA6 did not significantly (*p* > 0.05) differ for LAB values however, LAB values for these days were all significantly (*p* < 0.05) less than LAB values from fresh DA7 and DA8 tenderloins. From 60 h to 84 h slack time, LAB values from DA4, DA6 and DA7 all significantly (*p* < 0.05) increased, where DA5 did not change and DA8 values were significantly (*p* < 0.05) less. Similar to APC, DA4 tenderloins significantly (*p* < 0.05) increased in LAB values from fresh analysis to 132 h of slack time and was significantly (*p* < 0.05) greater than all other treatments at the end of the slacking period other than DA7 tenderloins, which started with the greatest APC values. Interestingly, DA5 and DA6 LAB values at 132 h slack time were significantly (*p* < 0.05) less than any other treatments at that time. Treatments DA4 and DA6 did not reach 10^1^ CFU/mL until 36 h slack time, where DA8 values were over 3 times higher. Overall, slack time nor days aged did not affect the development of lactic acid bacteria as no trend was found.

### 3.3. Pyschotropic Microbial Analysis

Results from the psychotropic plate count (PPC) microbial analysis are presented in Table 3. For this analysis no treatment reached 10^6^ CFU/mL, which was determined at the start of the study as the threshold for non-consumable products. At 36 h, DA4 tenderloins had significantly (*p* < 0.05) lower values than all other treatments at that time, however, no other treatments were different from each other. By 60 h slack time, no matter the treatment, there were no significant (*p* > 0.05) differences in PPC values throughout all treatments. At 84 h, DA5 was not significantly (*p* > 0.05) different from the values of DA6 tenderloins, however DA4 and DA5 values were both significantly (*p* < 0.05) greater than the tenderloins from DA7 and DA8, but not from each other. This trend continued into sampling at 108 h and 132 h, as DA4 and DA5 PPC values were significantly (*p* < 0.05) greater than any other treatments at those times. At 132 h both DA4 and DA5 treatments approached 10^6^ CFU/mL.

### 3.4. Storage Effect on pH

The storage time and age of chicken tenderloins following harvest greatly (*p* < 0.05) influenced the meat pH (Table 4). The meat pH was greatest initially (*p* < 0.05) on DA4, and the lowest (*p* < 0.05) as the duration of storage in refrigerated conditions extended through DA7 and DA8. 

### 3.5. Instrumental Surface Color

Surface color (L*) values for chicken tenderloins during the simulated shelf-life period are presented in Table 5. There was an interactive effect (*p* < 0.05) of age post-harvest and slack time on the surface color. Tenderloins were darkest (*p* < 0.05) on DA5 and lightest (*p* < 0.05) on DA8 of the simulated shelf-life period. As the duration of the storage period increased, the surface color of the chicken tenderloins became lighter (*p* < 0.05). 

## 4. Discussion

Aerobic and psychotrophic (except for DA4) microbial loads for the chicken tenderloins declined following marination with a water, salt, modified food starch, and monosodium glutamate. It is plausible that the addition of the marinade resulted in a lowering of the surface pH of the chicken tenderloins causing microorganism growth to be limited. Previous research comparing the microbial loads of raw chicken breasts to salted chicken breasts reported that the salted chicken breasts had reduced quantities of bacteria [6]. Additionally, the addition of the wet marinade could have had a diluting effect on the microbial load. Immediately after marinating, the chicken tenderloins were packaged and placed into a blast freezer (−24 °C). It has also been discussed that storage temperatures can alter bacterium growth [10]. The extremely cold storage temperatures can cause injury to bacterial cells which would then require favorable storage conditions (temperature) for repair [11].

Psychotrophs had the greatest microbial level throughout this study, likely due to the colder storage temperatures maintained throughout the storage periods. It appears that as the chicken tenderloins thawed, the psychotrophs were first to reach a favorable temperature for repair and grow much more rapidly than lactic acid or aerobic microbial organisms. The psychotropic bacteria tend to grow faster in colder temperatures (0 to 20 °C), while mesophilic bacteria grow faster in warmer temperatures (20 to 45 °C). A shelf-life experiment conducted compared how different temperatures (2 to 20 °C) affected the growth of psychotropic and mesophilic aerobic bacteria on portions of chicken breast [12]. The psychotropic microbial load was greater than the aerobic bacteria at the initial and last sampling periods, suggesting that colder temperatures during storage slow but do not eliminate microbial growth throughout shelf-life periods [12].

It has been described that at low refrigeration temperatures, psychotropic bacteria can dominate the competition and that mesophiles may survive the cold conditions but not grow [6]. Several studies have explained that the greatest influencer of microorganism growth is microbial competition [12,13]. *Pseudomonas* spp. is a very common specific spoilage organism that is an aerobic psychotroph. This bacterium is a common culprit in the poultry spoilage realm and the environment of this study favors bacteria like *Pseudomonads* and other aerobic pyschotrophs [8]. Rancid odors and slime production are signs of spoilage that are attributed to bacteria like *Pseudomonads* and can often be detected through sensory analytical methods [14]. According to a previous study, lactic acid bacteria did not create off odors equal to or greater than psychotropic bacteria, indicating that that sensory qualities are an important aspect of spoilage [4]. Moreover, when microbial counts exceed 10^8^ CFU/g, the production of slime associated with off odors is likely a contributing factor to the decomposition of muscle tissue [15].

The changes in postmortem muscle pH during the simulated storage period tend to agree with previous studies suggesting that changes in microflora, packaging atmosphere, and temperatures during storage period can impart changes to muscle pH [16,17,18]. Moreover, pH has been linked to influencing the many changes that occur in poultry meat quality which can include color, water-holding capacity, tenderness, and juiciness. In addition, surface color has been linked to the muscle pH of breast meat, with darker meat recording lower pH values [19]. The results within the current study agree with previous studies that higher pH values can produce a lighter surface color and lower pH values are associated with darker surface colors of chicken meat [20].

Surface color of meat is an important factor used to evaluate freshness or wholesomeness at the time of cooking. The surface color changes in lightness (L*) values that occurred during the storage period agree with previous studies [19,20]. Several factors have been reported to influence the surface color of chicken meat, including gender, age, and freezing conditions. In addition, broilers are harvested at younger ages resulting in less total myoglobin in the muscle tissue and a subsequent lighter surface color [21]. The surface color changes reported within the current study agree with previous studies where pH and storage temperature can impart light scattering as a function of protein denaturation [22].

## 5. Conclusions

In conclusion, spoilage organisms never achieved a spoilage threshold for this study of 6 log CFU/mL. The limited microbial growth and changes to surface color that occurred suggest that these findings could be utilized by the food service industry for chicken products that have been aged for longer periods post-slaughter prior to freezing. These results could bolster research used within the poultry industry in an effort to reduce waste/losses within the food service sectors, as products could be held under frozen, refrigerated, or even slacked conditions for a greater period of time without causing detrimental impacts to the freshness and wholesomeness of the chicken tenderloins. However, additional efforts are needed to identify methods of packaging and storage times that could further improve the quality attributes of taste and surface color of poultry products stored in refrigerated temperatures for extended periods.

## Figures and Tables

**Table 1:** Interactive impact (days aged ^1^ × slack time ^2^) on aerobic plate count values ^3^ of the chicken tenderloins during simulated food service shelf-life.

Days Aged	Slack Time (h)						
	Fresh	0 h	36 h	60 h	84 h	108 h	132 h
4 Days	1.42 ^klm^	0.662 ^n^	0.727 ^mn^	1.62 ^ijkl^	2.9 ^cd^	2.72 ^cdef^	2.96 ^bc^
5 Days	1.63 ^ijkl^	1.47 ^jkl^	0.971 ^lmn^	2.62 ^cdefg^	2.28 ^cdefghi^	2.38 ^cdefg^	1.95 ^ghijk^
6 Days	1.65 ^hijkl^	1.19 ^lmn^	2.54 ^cdefg^	2.34 ^cdefgh^	2.22 ^defghi^	1.51 ^jkl^	2.25 ^defghi^
7 Days	3.81 ^a^	3.62 ^ab^	2.04 ^fghijk^	2.95 ^bc^	1.52 ^jkl^	2.03 ^ghijk^	2.46 ^cdefg^
8 Days	2.82 ^cde^	2.58 ^cdefg^	2.14 ^efghij^	2.06 ^fghijk^	2.08 ^fghijk^	1.99 ^ghijk^	2.08 ^fghijk^
SEM	0.269	0.269	0.269	0.269	0.269	0.283	0.283

^1^ Frozen storage time (in days) of chicken following harvest. ^2^ Sampling period following fresh and frozen storage of packaged chicken tenderloins. ^3^ Colony forming units (CFU)/g of sampled chicken tenderloins. ^a–n^ LS Means lacking a common superscript differ (*p* < 0.05).

**Table 2:** Interactive impact (Days aged ^1^ × Slack time ^2^) lactic acid bacteria values ^3^ of the chicken tenderloins during simulated food service shelf-life.

Days Aged	Slack Time (h)						
	Fresh	0 h	36 h	60 h	84 h	108 h	132 h
4 Days	0.909 ^opqr^	0.34 ^qr^	1.14 ^nopq^	1.84 ^ijklmn^	3.02 ^cdefg^	2.69 ^efgh^	3.82 ^abc^
5 Days	0.997 ^opqr^	1.06 ^nopq^	1.97 ^hijklm^	1.72 ^jklmno^	1.06 ^nopqr^	3.77 ^abcd^	1.31 ^mnop^
6 Days	0.253 ^r^	0.496 ^pqr^	1.35 ^mno^	1.62 ^lmno^	2.31 ^ghijkl^	2.77 ^efgh^	1.39 ^mno^
7 Days	3.19 ^bcdef^	2.52 ^fghij^	1.65 ^klmno^	2.45 ^fghijk^	3.49 ^bcde^	2.38 ^ghijkl^	4.39 ^a^
8 Days	3.05 ^cdefg^	2.84 ^efg^	3.01 ^cdefg^	2.99 ^defg^	1.25 ^mnop^	3.99 ^ab^	2.61 ^fghi^
SEM	0.294	0.294	0.294	0.294	0.294	0.294	0.294

^1^ Frozen storage time (in days) of chicken following harvest. ^2^ Sampling period following fresh and frozen storage of packaged chicken tenderloins. ^3^ Colony forming units (CFU)/g of sampled chicken tenderloins. ^a–r^ LS Means lacking a common superscript differ (*p* < 0.05).

**Table 3:** Interactive impact (days aged ^1^ × slack time ^2^) of psychotropic plate count values ^3^ of the chicken tenderloins during simulated food service shelf-life.

Days Aged		Slack Time (h)					
	Fresh	0 h	36 h	60 h	84 h	108 h	132 h
4 Days	3.07 ^p^	3.16 ^op^	3.16 ^op^	3.69 ^hijk^	4.06 ^def^	4.95 ^b^	5.26 ^a^
5 Days	3.39 ^lmno^	3.15 ^op^	3.24 ^nop^	3.62 ^ijkl^	4.2 ^cd^	4.88 ^b^	5.07 ^ab^
6 Days	3.21 ^nop^	3.09 ^p^	3.27 ^mnop^	3.40 ^klmno^	3.80 ^efghi^	3.96 ^defgh^	4.4 ^c^
7 Days	3.63 ^ijkl^	3.53 ^ijklm^	3.39 ^lmno^	3.69 ^hijk^	3.71 ^ghij^	4.19 ^cd^	4.08 ^de^
8 Days	4.22 ^cd^	3.78 ^fghi^	3.47 ^jklmn^	3.65 ^ijkl^	3.64 ^ijkl^	3.99 ^defg^	4.1 ^cd^
SEM	0.118	0.118	0.118	0.118	0.118	0.118	0.118

^1^ Frozen storage time (in days) of chicken following harvest. ^2^ Sampling period following fresh and frozen storage of packaged chicken tenderloins. ^3^ Colony forming units (CFU)/g of sampled chicken tenderloins. ^a–p^ LS Means lacking a common superscript differ (*p* < 0.05).

**Table 4:** Interactive impact (days aged ^1^ × slack time ^2^) on pH ^3^ of the chicken tenderloins during simulated food service shelf-life.

Days Aged	Slack Time (h)						
	Fresh	0 h	36 h	60 h	84 h	108 h	132 h
4 Days	6.09 ^a^	6.06 ^a^	5.89 ^efghijk^	5.92 ^bcdefghijk^	5.93 ^bcdefghijk^	5.84 ^ijk^	5.94 ^abcdefghij^
5 Days	5.98 ^abcdefghi^	5.85 ^hijk^	5.90 ^bcdefghijk^	5.93 ^bcdefghijk^	5.83 ^ijk^	5.99 ^abcdefghi^	6.05 ^abcd^
6 Days	6.02 ^abcdefg^	5.86 ^ghijk^	5.89 ^defghijk^	5.87 ^fghijk^	6.05 ^abc^	6.02 abcdef	5.96 ^abcdefghij^
7 Days	5.89 ^cdefghijk^	5.84 ^ijk^	5.79 ^k^	6.02 ^abcdefg^	6.04 ^abcde^	5.96 ^abcdefghij^	5.83 ^ijk^
8 Days	5.80 ^jk^	5.94 ^abcdefhhijk^	6.01 ^abcdefgh^	6.02 ^abcdef^	6.02 ^abcdef^	5.90 ^bcdefghijk^	5.84 ^ijk^
SEM	0.073	0.073	0.073	0.073	0.073	0.073	0.073

^1^ Frozen storage time (in days) of chicken following harvest. ^2^ Sampling period following fresh and frozen storage of packaged chicken tenderloins. ^3^ pH is a measure of postmortem muscle pH. ^a–k^ LS Means lacking a common superscript differ (*p* < 0.05).

**Table 5:** Interactive impact (days aged ^1^ × slack time ^2^) on instrumental fresh surface lightness (L*) ^3^ of the chicken tenderloins during simulated food service shelf-life.

Days Aged	Slack Time (h)						
	Fresh	0 h	36 h	60 h	84 h	108 h	132 h
4 Days	54.03 ^ij^	59.44 ^abcdef^	57.96 ^cdefghi^	58.77 ^bcdefghi^	58.74 ^abcdefghi^	58.48 ^bcdefghi^	59.42 ^abcdef^
5 Days	54.74 ^k^	59.50 ^abcde^	57.86 ^defghi^	57.61 ^ghij^	57.89 ^defghi^	59.30 ^abcdefg^	58.30 ^bcdefghi^
6 Days	55.88 ^k^	54.97 ^k^	57.23 ^hij^	57.48 ^hij^	59.33 ^abcdefg^	59.92 ^ab^	57.35 ^hij^
7 Days	57.19 ^hij^	58.36 ^bcdefghi^	58.86 ^abcdefgh^	59.50 ^abcde^	57.15 ^hij^	58.45 ^bcdefghi^	59.67 ^abc^
8 Days	60.50 ^a^	59.69 ^abc^	59.66 ^abcd^	57.74 ^efghi^	57.62 ^fghij^	59.58 ^abcde^	60.50 ^ab^
SEM	0.665	0.665	0.665	0.665	0.665	0.665	0.665

^1^ Frozen storage time (in days) of chicken following harvest. ^2^ Sampling period following fresh and frozen storage of packaged chicken tenderloins. ^3^ L* values are a measure of darkness to lightness (larger value indicates a lighter color). ^a–k^ LS Means lacking a common superscript differ (*p* < 0.05).
